# Publications Received

**Published:** 1859-06

**Authors:** 


### PUBLICATIONS RECEIVED.

We have received a number of books, pamphlets, &c., which we hope to notice in the next number.

We assure those who have thus favored us, and who have been apparently neglected, that it is not from any want of appreciation of their courtesy, but from an actual inability for several months, to give the attention to our journal which it requires, and which we hope to be able to give it in the future.

